# Translational albumin nanocarrier caging photosensitizer for efficient cancer photodynamic therapy

**DOI:** 10.3389/fbioe.2023.1132591

**Published:** 2023-02-01

**Authors:** Jie Luo, Zhijun Miao, Xinglong Huang, Yifan Yang, Ming Liu, Gang Shen, Tao Yang

**Affiliations:** ^1^ Jiangsu Key Laboratory of Neuropsychiatric Diseases, College of Pharmaceutical Sciences, Soochow University, Suzhou, China; ^2^ Department of Urology, Dushu Lake Hospital Affiliated to Soochow University, Suzhou, China

**Keywords:** human serum albumin, photosensitizer, target delivery, photodynamic therapy, bladder cancer

## Abstract

It still remains a great challenge to efficiently treat malignant cancers which severely threaten human health. Photodynamic therapy (PDT) as a localized therapeutic modality has improved the therapeutic efficacy via chemical damage through reactive oxygen species (ROS). However, their efficacy is severely hampered by insufficient targeted delivery of photosensitizers owing to the lack of suitable carrier with facile preparation process and the clinical applicability. Herein, we applied clinically approved human serum albumin as the nanoreactor to encapsulate photosensitizers Chlorin e6 (Ce6) for enhancing their tumor accumulation and subsequently potent PDT effect against bladder cancer models. Albumin-loaded Chlorin e6 nanoparticles (CA-NPs) with rational nanoscale size exhibit increased reactive oxygen species production and excellent resistance to photobleaching. Moreover, CA-NPs could be efficiently internalized by tumor cells and locate in the lysosome, while they rapidly translocate to cytosol after irradiation to induce remarkable cytotoxicity (IC_50_ ∼5.8 μg/ml). Furthermore, CA-NPs accumulate effectively in tumor tissue to afford total eradication of murine bladder tumor after single injection. More importantly, we also evidence the superior PDT effect in fresh human bladder tumor tissues via abundant reactive oxygen species generation and subsequent cell apoptosis. These findings demonstrate that human serum albumin acts as a universal tool to load small organic photoactivatable molecule with remarkable effectiveness and readiness for clinical translation.

## 1 Introduction

Aggressive cancers have caused leading human death during the past few years, significant challenges still exist to explore effective solutions against various types of cancers to prolong the survival of cancer patients (Sung et al., 2021). Although chemotherapy and surgery are commonly applied in clinic settings (Nencioni et al., 2018; Joshi and Badgwell, 2021), the overall outcome remains relatively low due to their intrinsic limitations such as severe side effects, drug resistance, poor targeting ability, and rapid recurrence, finally leading to the failure of cancer treatment (Patel et al., 2020; Labrie et al., 2022).

Photodynamic therapy (PDT) as a localized therapeutic modality which activates photosensitizers specifically within targeted organs upon light irradiation to induce chemical damage *via* reactive oxygen species (ROS) has been used in clinic against various cancers including superficial skin lesions and lung cancers for over 40 years (Li et al., 2020; Pham et al., 2021). However, existing PDT solutions suffer from poor blood circulation, limited tumor accumulation, and relatively short excitation wavelength of photosensitizers, thus severely hampering the *in vivo* therapeutic efficiency of PDT (Yang et al., 2021). With the development of nanotechnology, extensive efforts have been made to develop efficient nanocarriers incorporated with photosensitizer to improve their targeting ability (Xie et al., 2021). For example, polymeric materials with tunable surface properties (e.g., charge, size, and ligand installing) were applied to load photosensitizers for enhanced tumor delivery (Zhen et al., 2019; Zhu et al., 2020; Yi et al., 2021). Also, photosensitizer could be covalently conjugated to the surface of nanocarriers (e.g., inorganic nanomaterials, silica materials) *via* chemical modification to increase their tumor accumulation (Wang et al., 2019; Zheng et al., 2021). However, the potential clinic translation of these solutions is distinctly restricted by complex fabrication process and unapproved raw materials. Therefore, additional attentions should be focused to develop suitable nanocarriers with readiness for clinical translation and convenient preparation process.

Abraxane that is composed of human serum albumin (HSA) and paclitaxel with size around 130 nm has achieved huge success in clinic to reduce the side effects in diverse cancers (Yardley, 2013; Gianni et al., 2018). Inspired by this approach, we and other groups recently demonstrated that albumin and other proteins could be applied as a nanoreactor to allow the growth of theranostic inorganic nanocrystals such as tellurium (Yang et al., 2017), gadolinium oxide (Zhou et al., 2017; Lv et al., 2018), copper sulfide (Yang et al., 2016), manganese oxide (Li et al., 2022; Zhai et al., 2022) inside albumin or transferrin nanocage *via* precipitation reaction or redox reaction, exhibiting superior tumor targeting ability and high drug loading efficiency. However, incorporating small molecule photosensitizer inside albumin nanoreactor remains largely unexplored with limited solutions. Herein, we employed clinically approved HSA as the carrier to encapsulate photosensitizer Chlorin e6 (Ce6) *via* well-defined precipitation inside albumin nanocage for targeted delivery of Ce6 and potent PDT effect against murine bladder cancer and restricted human bladder tumor tissue. The albumin-loaded Ce6 nanoparticles (CA-NPs) with suitable size distribution showed improved photostability and photoactivity, as well as excellent resistance to photobleaching. Moreover, CA-NPs efficiently accumulated in tumor site and rapidly generate ROS after irradiation *in vivo*, resulting in an increased cytotoxicity to eradicate murine bladder cancer after single injection. Importantly, CA-NPs also exhibited abundant ROS production in freshly restricted human bladder tumor tissue to induce effective cell apoptosis upon irradiation. These results demonstrate the HSA as a universal nanocarrier to incorporate small molecule drugs with enhanced effectiveness and clinical applicability.

## 2 Materials and methods

### 2.1 Synthesis and characterization of CA-NPs

For the preparation of CA-NPs, Ce6 (Macklin, China) was dissolved in aqueous solution of pH 12 with concentration of 1 mg/ml. HSA (CSL Behring GmbH) was diluted to 10 mg/ml. Subsequently, Ce6 solution was added to HSA solution in a 5:1 (v/v) ratio under stirring. After the mixture was stirred for 5 min, hydrochloric acid (HCl) was used to adjust the pH to 5.5. The mixture was then stirred for another 2 h at room temperature. The resulting CA-NPs was transferred into Millipore and centrifuged at 2000 rpm for 10 min to remove free Ce6, and stored at PBS for further use.

For the characterization of CA-NPs, the morphology and size of CA-NPs were characterized by transmission electron microscope (Hitachi HT-7700, Japan) and dynamic light scattering (Malvern Zetasizer ZS90, England). The absorbance of CA-NPs was measured by UV-vis spectra (UV-2600, Shimadzu).

For determination of drug loading of CA-NPs, CA-NPs were prepared and purified as above, and concentration of Ce6 wad measured by ultraviolet spectrometer. Then, CA-NPs solution was freeze-dried and recorded the weight. The drug loading was calculated by the formula: loading efficiency (%) = weight of Ce6 encapsulated in CA-NPs/weight of CA-NPs × 100%.

### 2.2 ROS production photostability, and drug release

Free Ce6 and CA-NPs were diluted with the ultrapure water to 10 μg/ml, and then irradiated at the excitation wavelength of 660 nm with the power density of 0.15 W/cm^2^ (FS-Optics, China). Simultaneously, the temperature was monitored with an electronic thermometer for 5 min. Besides, the stability of them were measured by UV-vis spectrophotometer under the same condition. The absorbance at the wavelength of 660 nm was monitored within 5 min.

1,3-diphenylisobenzofuran (DPBF) was employed as singlet oxygen probe to detect the generation of singlet oxygen. CA-NPs and free Ce6 with the concentration of 1.0 μg/ml (Ce6) were mixed with 30.0 μM DPBF under vigorous stirring, followed by 5 min irradiation (660 nm laser, 0.15 W/cm^2^). Meanwhile, the absorbance of DPBF at 420 nm was monitored by UV-vis spectrophotometer during the irradiation.

The drug release behaviors of Ce6 from CA-NPs were evaluated using the dialysis method. CA-NPs with concentration of 100 μg/ml Ce6 was dialyzed in various media including pH 5.0 buffer, pH 6.5 buffer and pH 7.4 buffer and the same concentration of free Ce6 was served as the control group. The various formulations (1.0 ml for each sample) were separately added to dialysis bags incubated in 50 ml of release buffer. Then, the *in vitro* releases were performed in a contrast temperature oscillator shaker at 37°C. Each 0.5 ml sample was taken at 0.5, 1, 2, 4, 8, 12, 24, and 48 h with the replacement of an equal volume of fresh medium. The concentrations of Ce6 were measured using microplate reader (TECAN, Switzerland) at the wavelength of 660 nm.

### 2.3 Cell lines and cell culture

MB49-bladder carcinoma cells (ATCC, United States) were cultured in Dulbecco’s modified Eagle’s medium (DMEM, Gibco, America) supplemented with 10% fetal bovine serum (FBS, Bovogen Biological, Australia), 100 U/mL penicillin (Gibco, America) and 100 μg/ml streptomycin (Gibco, America). All the cells were grown in an atmosphere with 37°C and 5% CO_2_.

### 2.4 Cellular uptake and endocytic pathways

To quantify the cellular uptake of Ce6, murine MB49 cells were seeded in 2 × 10^6^ cells per well in 6-well plates and treated with PBS, free Ce6 and CA-NPs for 24 h at concentrations of 10 μg/ml (Ce6), respectively. Then, the cells were washed, harvested and suspended in a 2% bovine serum albumin in PBS solution and analyzed *via* flow cytometry (BD FACSVerse, America) using a 640 nm excitation laser and 780/15 filter configuration.

To determine the endocytic pathways of CA-NPs, MB49 cells were seeded in 6-well plates and treated with clathrin-dependent endocytosis inhibitor chlorpromazine (10 μg/ml), caveolin-dependent endocytosis inhibitor nystatin dihydrate (5.0 μg/ml) and macropinocytosis inhibitor amiloride (100.0 μg/ml) for 2 h. Then, CA-NPs (10.0 μg/ml) were added into the culture medium for further 24 h incubation, followed by the same procedure as used for cellular uptake. The data were processed and analyzed using FlowJo (v.10.5).

### 2.5 Cell viability assay

MTT assay (Macklin, China) was used to examine the cytotoxicity of free Ce6 and CA-NPs against MB49 cells. MB49 cells were seeded in a 96-well plate at the density of 5 × 10^3^ cells per well and cultured overnight. After that, free Ce6 and CA-NPs with the concentration of 0, 2, 4, 6, 8, 10 and 12 μg/ml were added into the plate to co-incubate with cells for 24 h. Then, the medium was removed and the cells were washed with PBS, followed by adding fresh medium and 5 min irradiation (660 nm, 0.15 W/cm^2^). After another 24 h incubation, the cell viability was evaluated using MTT assay. After 4 h of incubation at 37°C and 5% CO_2_, the absorbance at 490 nm was measured by microplate reader (TECAN, Switzerland).

### 2.6 Intracellular distribution, ROS detection and lysosomal disruption

MB49 cells were seeded in the glass-bottom confocal dishes and treated with 5 μg/ml of rhodamine B-labeled CA-NPs (CA-NPs-RB) for 24 h, Then, the cells were washed, irradiated for 5 min (660 nm, 0.15 W/cm^2^) and stained with 100 nM Lysotracker Green DND-26 (Beyotime, China) and Hoechst33342 (Beyotime, China). For ROS detection, 2′,7′-dichlorodihydrofluorescein diacetate (DCFH-DA) was used as singlet oxygen probe to examine the generation of ROS. After incubation with free Ce6 or CA-NPs (5 μg/ml), the cells were treated with 1 µM of DCFH-DA (Beyotime, China) for 30 min before irradiation. Then, the cells were irradiated for 5 min (660 nm, 0.15 W/cm^2^), followed by staining with Hoechst33342 (1 μg/ml) for 8 min. To observe the disruption of lysosomal membranes, acridine orange (AO) was used as an intracellular indicator of acidic organelle integrity in cells. MB49 cells were treated with PBS, free Ce6 and CA-NPs at 5 μg/ml Ce6 for 12 h. Then, the cells were irradiated for 5 min (660 nm, 0.15 W/cm^2^) and 2 mM vitamin C was incubated with cells before irradiation, followed by staining with AO working solution (5 μM) for 20 min, the cells were washed and further washed three times with PBS before being observation. The intracellular fluorescence was observed by confocal laser scanning microscope (CLSM) and the median fluorescence intensity (MFI) of cells was counted by ImageJ.

### 2.7 Measurement of mitochondrial membrane potential

Mitochondrial membrane potential was recorded by using the fluorescent indicator 5,5′,6,6′-tetrachloro-1,1′,3,3′-tetraethyl-imidacarbocyanine iodide (JC-1) molecular probes (Beyotime, China). MB49 cells treated with CA-NPs were irradiated for 5 min with the power density of 0.15 W/cm^2^. After further incubation for 30 min, the cells were incubated with JC-1 working solution for 20 min and Hoechst33342 (1 μg/ml) for 8 min. Then, the cells were washed and observed by CLSM within 30 min. The MFI of JC-1 aggregates and monomers were counted by ImageJ.

### 2.8 Cell apoptosis detection

The proportion of cell apoptosis induced by CA-NPs was detected by Annexin V FITC/PI Apoptosis kit (Multi Science, China). MB49 cells treated with CA-NPs and irradiated (0.15 W/cm^2^, 5 min) were harvested and suspended with binding buffer. Then, the cells were stained with Annexin V-FITC/PI working solution for 5 min and subjected to flow cytometry detection.

### 2.9 *In vivo* and *ex vivo* distribution

6–8 weeks C57BL/6J mice were subcutaneously injected with 1 × 10^6^ MB49 cells (50 µl) to establish subcutaneous bladder tumor models. When the tumor volume reached to 100 mm^3^ (tumor volumes were measured by a vernier caliper and were calculated as following: V_tumor_ = 0.5 × length × width^2^), the mice were divided randomly into two groups (n = 3) and then were respectively injected with Ce6 and CA-NPs (5.0 mg/kg) *via* tail vein, followed by *in vivo* imaging at the excitation of 660 nm using IVIS spectrum (Perkin Elmer, United States) at 0, 2, 6, 12, 24, 48 h. For *ex vivo* distribution study, the mice bearing MB49 tumor models were sacrificed after 12 h post-injection of CA-NPs or free Ce6, then the major organs including heart, liver, spleen, lung, kidney and tumor were utilized for *ex vivo* imaging. The fluorescence intensities were obtained by Living Image software (4.5).

### 2.10 ROS production in tumor tissues

C57BL/6J mice bearing MB49 tumors were divided randomly into six groups and injected with PBS, free Ce6 and CA-NPs *via* tail vein at the dose of 5.0 mg/kg, respectively. Then the mice were suffered with or without 660 nm irradiation (0.15 W/cm^2^, 5 min) after 12 h post-injection. In addition, DCFH-DA (10 μM) was prepared for intratumoral injection half an hour before irradiation. The mice were sacrificed after 1 h post-irradiation, then the MB49 tumors were cut into slices by freezing microtome for DCFH-DA staining assay. The tumors were washed with PBS three times, then treated with Hoechst33342 (1 μg/ml) for 10 min. We further acquired the images of slices using CLSM.

### 2.11 *In vivo* infrared imaging and anticancer efficacy

C57BL/6J mice bearing MB49 tumors were intravenously injected with PBS, free Ce6 and CA-NPs at the dose of 5.0 mg/kg. Then, the mice were irradiated at 0.15 W/cm^2^ for 5 min at 12 h post injection. Temperature of the tumor region under irradiation were monitored using an infrared camera during 5 min. The temperature of tumor region was measured by Smartview4.3.

C57BL/6J mice bearing MB49 tumors were randomly divided into six groups and treated with PBS, PBS plus irradiation, free Ce6, free Ce6 plus irradiation, CA-NPs, CA-NPs plus irradiation at the dose of 5.0 mg/kg on day 0, followed by 660 nm laser after 12 h post-injection. The tumor size and body weight were measured during the next 21 days. Then the mice were scarified on the 21^st^ day and tumor weight were measured by electronic balance (Sartorius, Germany).

### 2.12 *Ex vivo* ROS generation and apoptotic analysis of resected human bladder tumor tissue

Patients provided consent for the use of biospecimens for research as approved by the Clinical Trial Ethics Committee of Dushu Lake Hospital Affiliated of Soochow University. Within 30 min of surgical resection at Dushu Lake Hospital Affiliated of Soochow University, human bladder tumor tissues were submerged in Roswell Park Memorial Institute 1640 media (Gibco, America) supplemented with 10% FBS, 1% insulin-transferrin-selenium, 1% GlutaMAX (Gibco, America), and 1% penicillin-streptomycin (Gibco, America) and divided into twelve sections, followed by intratumoral injection with PBS and CA-NPs at the dose of 1 μg Ce6 for another incubation of 1 h. Then some of them were irradiated at the density of 0.15 W/cm^2^ for 5 min. After another 1 h incubation, half of these samples were cut into slices by freezing microtome for immunofluorescence staining of TUNEL assay. And the others were dissociated with 1 mg/ml collagenase II and 0.1 mg/ml DNase I in 1640 medium at 37°C for 45 min after irradiation. The single cells were harvested and counted, and 1 × 10^6^ cells suspended in staining buffer were used for TUNEL staining measured by flow cytometry.

### 2.13 Statistical analysis

All experiments were carried out at least three times and data were presented as mean ± standard deviation. The differences between two groups were evaluated using Student’s t-test, whereas the statistical analysis of multiple groups was performed using the one-way ANOVA in GraphPad Prism 8. Significant differences are indicated as *p < 0.05, **p < 0.01, ***p < 0.001.

## 3 Results and discussion

### 3.1 Preparation and characterization of CA-NPs

**FIGURE 1:**
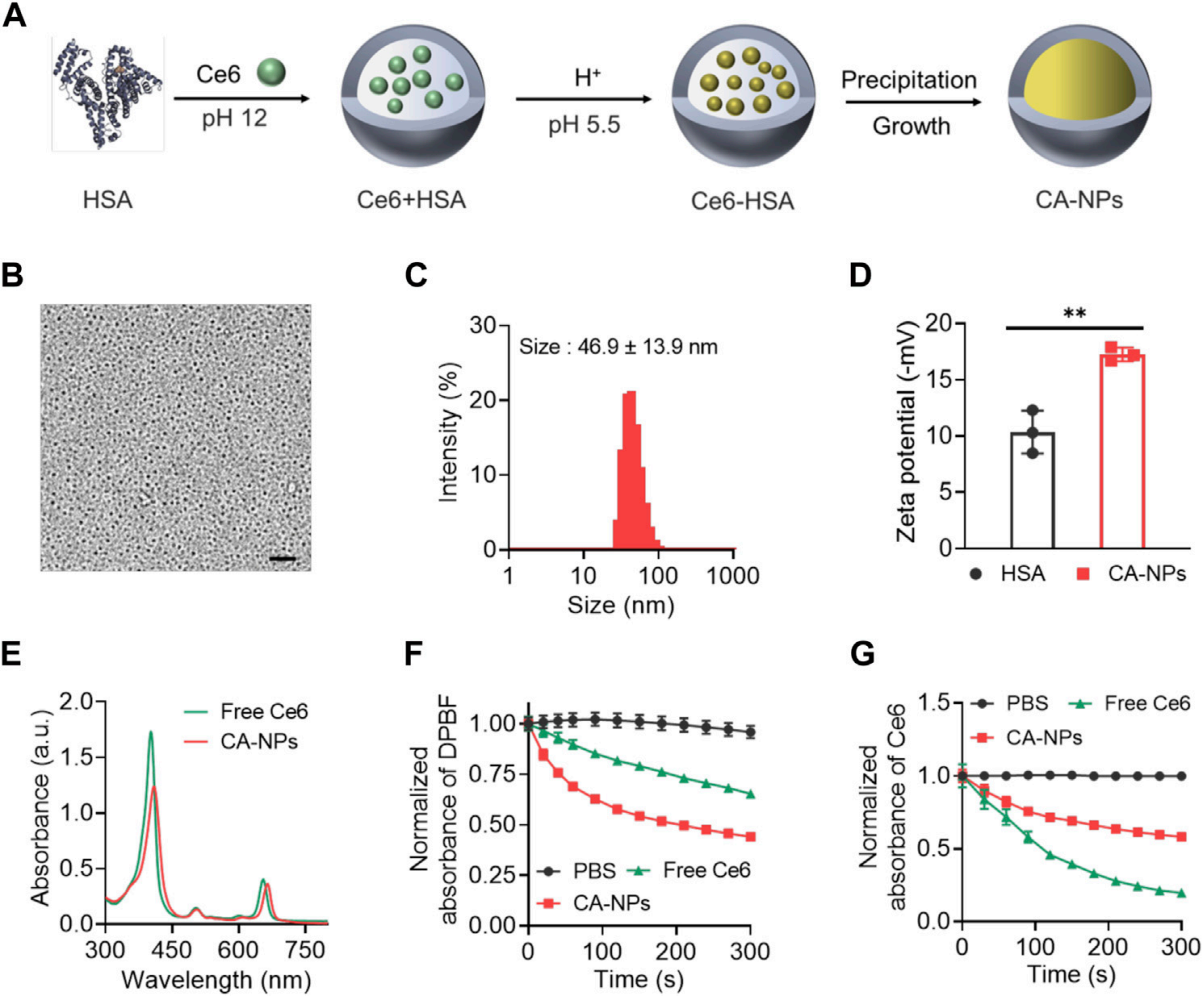
Synthesis and characterization of CA-NPs **(A)**. Preparation of CA-NPs through Albumin Nanoreactor. **(B)** Transmission electron microscope image of CA-NPs. Scale bar: 200 nm. **(C)** Size distribution of CA-NPs. **(D)** Zeta potential of HSA and CA-NPs. (n = 3; **p < 0.01) **(E)** UV-Vis absorption spectrum of free Ce6 and CA-NPs at the concentration of 5 μg/ml Ce6. **(F)** ROS generation of free Ce6 and CA-NPs under 5 min irradiation (660 nm, 0.15 W/cm^2^) using DPBF as a probe. **(G)** UV-Vis absorbance of PBS, free Ce6 and CA-NPs under irradiation during 5 min.

In a typical synthesis process, HSA was applied as a nanoreactor to allow the precipitation reaction of Ce6 within the hollow albumin nanocage. Firstly, Ce6 dissolved in basic solutions (pH 12) was mixed with HSA to form the Ce6-HSA complex, and the pH of the solution was subsequently adjusted to 5.5 using HCl buffer with ionized Ce6 molecular tuning to be non-ionized, followed by vigorous stirring under room temperature for 4 h for the growth of CA-NPs (Figure 1A). Then the obtained solution was purified *via* ultrafiltration for further use. As shown in Figure 1B, the round-like CA-NPs exhibited uniform size of ∼18.5 ± 3.2 nm upon the observation using transmission electron microscopy with the drug loading of 9.6 ± 1.1%, while the light scattering diameter of CA-NPs was slightly larger (∼46.9 nm with the PDI value of 0.26) owing to the outer shell (Figure 1C). Furthermore, the diameter of CA-NPs remained relatively unchanged in aqueous solution, PBS and culture medium during 1 week, indicating the superior stability (Supplementary Figure S1). Meanwhile, CA-NPs was negatively charged after loading Ce6 into the HSA nanocage (Figure 1D). Next, typical absorbance of Ce6 at 660 nm and 410 nm confirmed the successful incorporation of Ce6 in CA-NPs, and the CA-NPs showed obvious red-shifted peak of Ce6, suggesting the *J*-type aggregation of Ce6 inside HSA nanocage (Figure 1E). Drug release behavior plays the vital role in their biological performance, and thus the drug releases from CA-NPs were evaluated in response to physiological environment and acidic tumor microenvironment. As shown in Supplementary Figure S2, the accumulative release of CA-NPs within 48 h was less than 10% due to the hydrophobic property of the Ce6, indicating a preferable ability to minimize their undesirable release during blood circulation.

According to the clinical setting for *in vivo* PDT against malignant diseases, we choose 0.15 W/cm^2^ for 660 nm irradiation which is well tolerated for human and would not cause any significant side effects (Katsikanis et al., 2020). To measure photothermal effect and ROS production of CA-NPs under irradiation at human tolerance and clinically approved power density of 0.15 W/cm^2^ with the wavelength of 660 nm, DPBF as applied as a specific probe to detect the ROS generation via monitoring the absorbance at 420 nm, which possesses a highly specific reactivity towards singlet oxygen forming an endoperoxide 1,2-dibenzoylbenzene, resulting a decrease absorbance at 420 nm. As shown in Figure 1F, CA-NPs exhibited higher ROS production as compared to free Ce6 without any increase of solution temperature (Supplementary Figure S3) and the ROS production of CA-NPs group was 1.6 times higher than that in the free group. Moreover, we then tested the photobleaching of CA-NPs and free Ce6 during irradiation for 5 min. Similarly, CA-NPs showed enhanced resistance against photobleaching as compared to free Ce6. It was indicated that the absorbance of free Ce6 underwent a sharp decrease within 5 min due to the rapid photobleaching of free Ce6 under irradiation. However, the absorbance of CA-NPs still exhibited sufficient absorbance owing to significant improvement of photostability of Ce6 in CA-NPs (Figure 1G). Collectively, the increased photostability and photoactivity confer the potential of CA-NPs to yield robust photodynamic therapeutic effect against intractable cancers both *in vitro* and *in vivo*.

### 3.2 Cellular uptake, endocytic pathway and subcellular translocation

**FIGURE 2:**
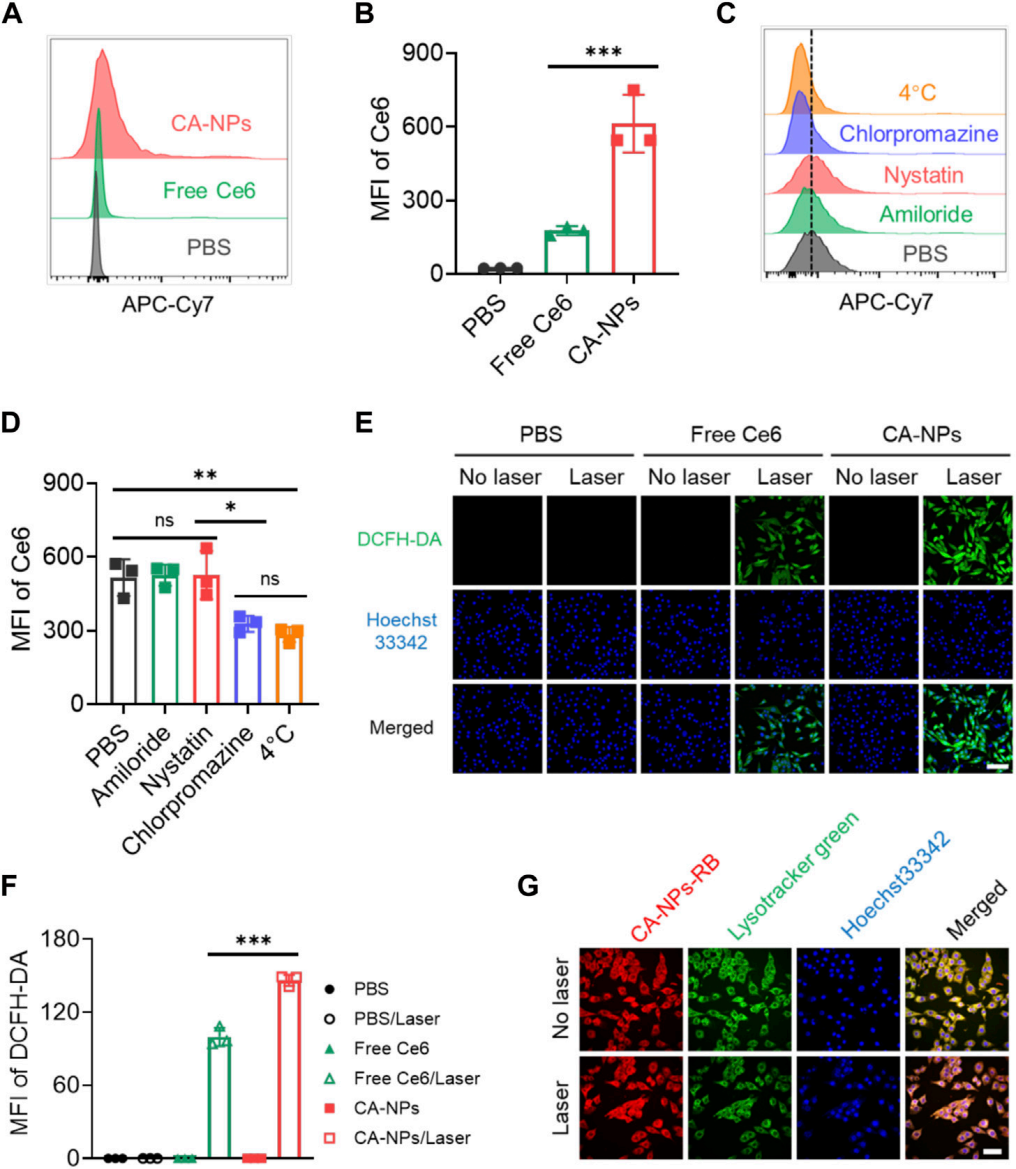
Cellular uptake, endocytic pathway and subcellular translocation of CA-NPs. **(A,B)** Flow cytometric quantification of the uptake of PBS, free Ce6 and CA-NPs at the dose of 10 μg/ml by MB49 cells for 24 h **(C,D)** Flow cytometric quantification of the uptake of CA-NPs by MB49 cells treated with PBS, amiloride, filipin and chlorpromazine at 37°C, and PBS at 4 °C. **(E,F)** Intracellular ROS generation and MFI of free Ce6 and CA-NPs under 660 nm irradiation (0.15 W/cm^2^, 5 min) or not at the dose of 5 μg/ml detected by DCFH-DA probe. Scale bar: 100 μm. **(G)** Subcellular distribution of CA-NPs labelled with rhodamine B under irradiation or not. Scale bar: 50 μm. (n = 3; ns: no significance, *p < 0.05, **p < 0.01, ***p < 0.001).

To evaluate the ability of CA-NPs to be internalized by cancer cells, we applied flow cytometry to quantitatively measure the cellular uptake of CA-NPs in murine bladder cancer cell lines (MB49) using free Ce6 as a control. At 24 h post-incubation of CA-NPs and free Ce6 at the dose of 5 μg/ml with MB49 cells, significantly increased internalization amount was observed in CA-NPs and was 3.5-fold higher than that of free Ce6 group (Figures 2A,Figures 2B). Moreover, chlorpromazine as an inhibitor of clathrin-dependent pathway distinctly decreased the cellular uptake of CA-NPs, indicating the internalization was depended on clathrin pathway. Meanwhile, when incubated CA-NPs with MB49 cells under 4°C, the intracellular amount of CA-NPs was declined to 55% as compared to PBS group (Figures 2C,D), confirming the energy also participated in the cellular uptake behavior. Next, we investigated the intracellular ROS production of CA-NPs upon irradiation using DCFH-DA as a probe. Both CA-NPs and free Ce6 showed negligible ROS production without irradiation, while durable green fluorescence from CA-NPs group emerged upon irradiation (Figure 2E) and was 1.6-fold higher than that of free Ce6 group (Figure 2F), reasonably owing to enhanced photoactivity and increased cellular uptake. We further verified the capacity of CA-NPs to induce lysosomal disruption under irradiation using AO staining. The acidic lysosomes in MB49 cells treated with PBS or PBS plus irradiation exhibited overlapped orange fluorescence between red and green fluorescence. And the red fluorescence was significantly decreased after the treatment of free Ce6 and CA-NPs upon irradiation. However, red fluorescence could be clearly observed in the MB49 cells pre-incubated with ROS scavenger (vitamin C), indicating that ROS production from CA-NPs upon irradiation participate in the disruption of lysosomal membranes upon irradiation (Supplementary Figure S4), which is in favor of the translocation of released Ce6 into cytoplasm for enhancing cytotoxicity. Considering the intracellular target of ROS to damage cancer cells is nucleus, we then investigated the subcellular location of CA-NPs with and without irradiation. MB49 cells were firstly labelled with Lysotracker Green and Hoechst33342 to distinguish lysosome and nucleus. At 6 h post-incubation of CA-NPs labelled with rhodamine B, preferable co-localization rate (92.1%) of CA-NPs with lysosome was observed as indicated by the merged yellow fluorescence, and CA-NPs rapidly translocated to cytosol upon irradiation with decreased co-localization rate (54.3%) *via* photo internalization effect (Figure 2G), which is favorable for causing cell damage.

### 3.3 Cytotoxicity and apoptotic level of CA-NPs

**FIGURE 3:**
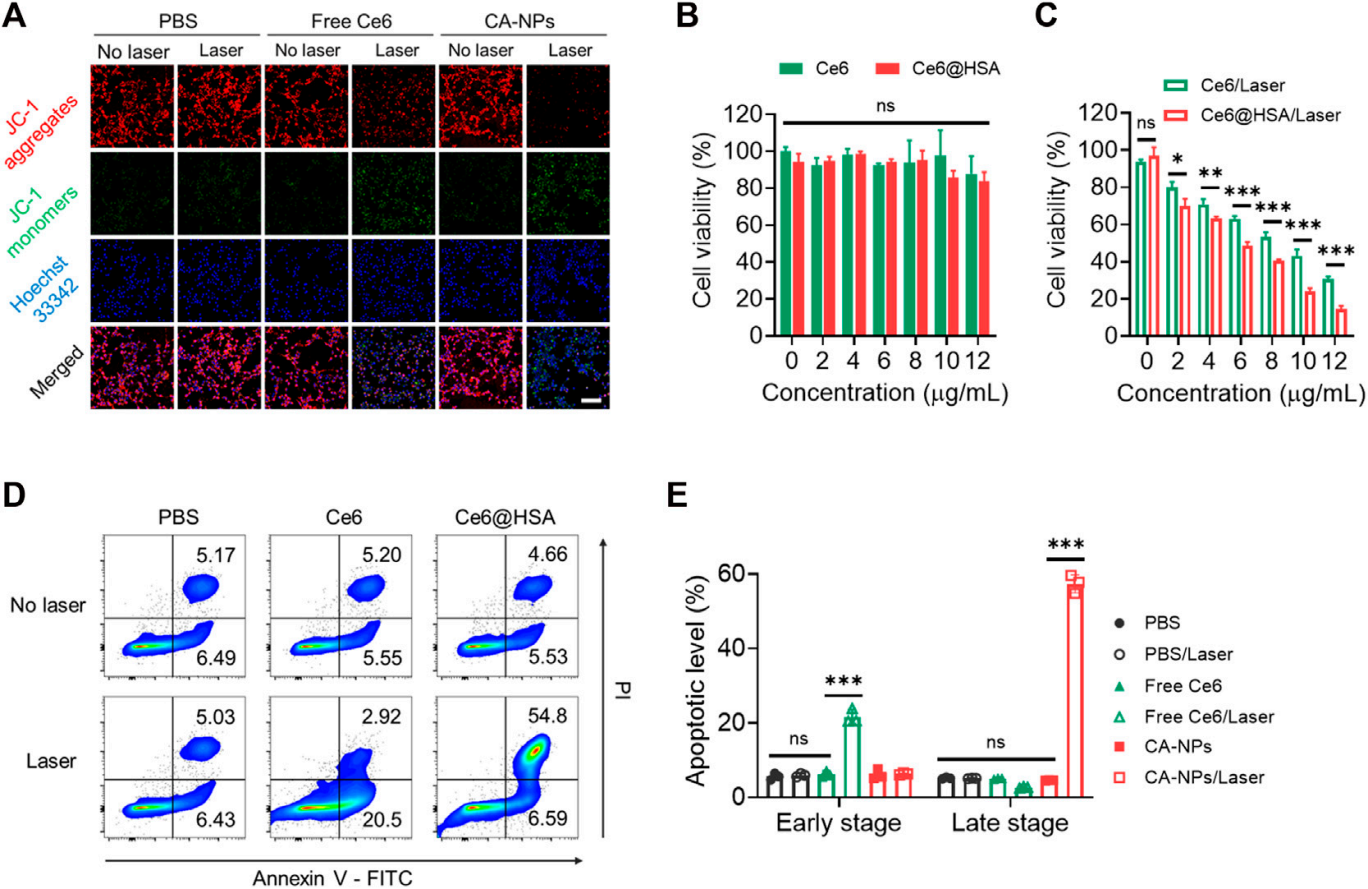
Cytotoxicity and tumor-killing mechanism of CA-NPs. **(A)** Mitochondrial membrane potential of MB49 cells treated with PBS, free Ce6 and CA-NPs at the dose of 5 μg/ml Ce6 detected by JC-1 assay. Scale bar: 100 μm. **(B,C)** Cell viability of MB49 cells treated with free Ce6, CA-NPs under 660 nm irradiation or not (0.15 W/cm^2^, 5 min). **(D,E)** Flow cytometric measurement of the apoptotic percentages of MB49 cells after 24 h incubation with PBS, free Ce6 and CA-NPs under irradiation or not. (n = 3; ns: no significance, *p < 0.05, **p < 0.01, ***p < 0.001).

To evaluate the cytotoxicity of CA-NPs against MB49 cells, we firstly applied the JC-1 assay to test mitochondrial membrane potential (ΔΨm) that correlated with cytotoxicity under oxidized stress conditions *via* ROS, in which JC-1 emits strong red fluorescence for high ΔΨm and green fluorescence for low ΔΨm, respectively. Ignorable change of red fluorescence was observed from the tumor cells treated with CA-NPs in the absence of irradiation, while CA-NPs showed minimal red fluorescence and emitted strong green fluorescence upon light exposure as compared to that of saline group and free Ce6 group (Figure 3A, Supplementary Figure S5), suggesting that CA-NPs possess a sharply decreased ΔΨm that reveals robust cytotoxicity against tumor cells. Subsequently, CA-NPs induced considerable photodynamic effect against MB49 cells with the IC_50_ value of 5.8 μg/ml upon irradiation, while free Ce6 with relatively decreased cellular uptake showed 1.5-fold higher IC_50_ value of 8.9 μg/ml (Figure 3C). Meanwhile, both free Ce6 and CA-NPs failed to arouse any toxicity in the absence of irradiation (Figure 3B). Next, Annexin V assay were used to measure the apoptotic levels of CA-NPs. As indicated in Figure 3D, CA-NPs led to significantly elevated apoptosis, especially the late stage, upon irradiation as compared with free Ce6 group (Figure 3E), further evidencing the potent PDT effect against MB49 cells.

### 3.4 *In vivo* targeting ability of CA-NPs

**FIGURE 4:**
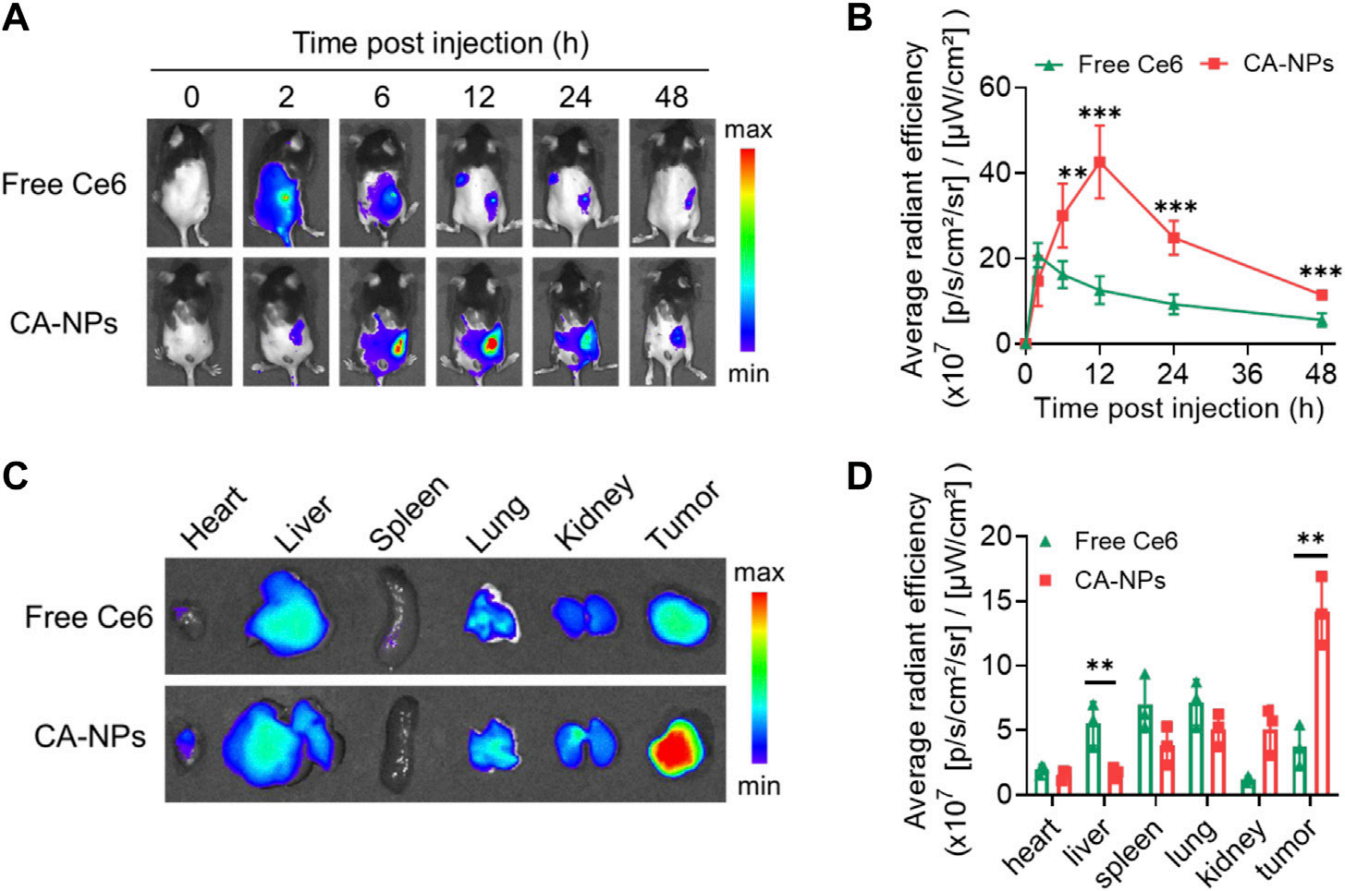
*In vivo* targeting ability of CA-NPs **(A,B)** *In vivo* fluorescence images and fluorescence intensities at tumor sites of MB49 tumor-bearing mice with intravenous injection of free Ce6 and CA-NPs at the dose of 5.0 mg/kg during 48 h (n = 4; **p < 0.01, ***p < 0.001). **(C,D)** *Ex vivo* fluorescence images and fluorescence intensities of various tissues extracted at 12 h from MB49 tumor-bearing mice with intravenous injection of free Ce6 and CA-NPs. (n = 3; **p < 0.01).

To unravel the *in vivo* targeting ability of CA-NPs, we firstly constructed the murine bladder tumor model via subcutaneous injection of MB49 cells (1 × 10^6^) into C57BL/6J mice. When the tumor volume reaches 100 mm^3^, CA-NPs at the dose of 5.0 mg/kg were intravenously injected into mice bearing MB49 bladder tumor model using free Ce6 as a control. Then, the mice were observed using IVIS imaging system to track the targeting behavior of CA-NPs by fluorescent signals from Ce6. Firstly, free Ce6 distributed quickly in the whole mice and were also cleared out of mice bearing MB49 bladder tumor model rapidly with minimal fluorescent signals observed at 12, 24 and 48 h post-injection (Figure 4A). While CA-NPs with suitable size distribution gradually accumulated in the tumor site (Figure 4A), possibly owing to enhanced permeability and retention effect as well as receptor-mediated active targeting ability of albumin (such as GP60). The highest amount of CA-NPs in the tumor site was detected at 12 h post-injection and was 2.8-fold higher than that of free Ce6 group (Figure 4B). We further collected major organs including heart, liver, spleen, lung, kidney and tumors to quantitatively measure the fluorescent intensities of CA-NPs. As shown in Figure 4C, although CA-NPs distributed in liver and kidney, significantly increased accumulation of CA-NPs was found in tumor tissues and was 3.0-fold and 2.8-fold higher than that of liver and kidney, respectively (Figure 4D), further confirming the superior targeting ability of CA-NPs for *in vivo* PDT effect.

### 3.5 *In vivo* anti-tumor effect of CA-NPs

**FIGURE 5:**
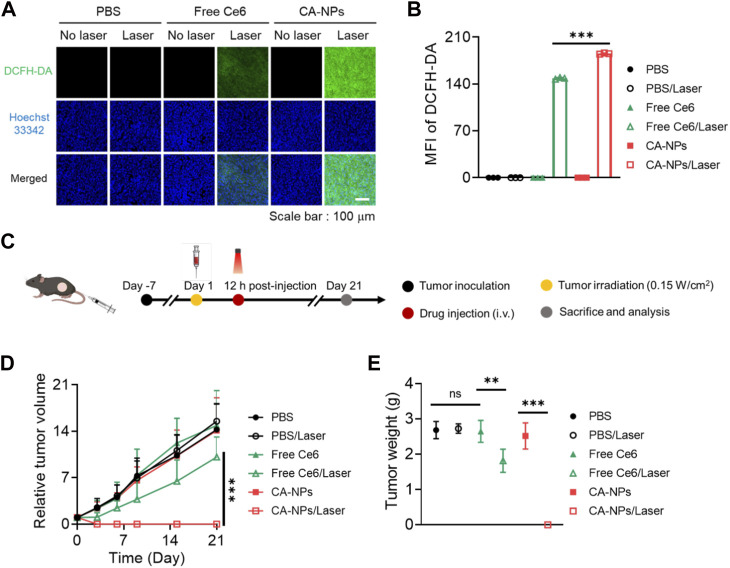
*In vivo* anti-tumor effect of CA-NPs. **(A,B)** Confocal images and fluorescence intensities of the MB49 tumor from the mice treated with PBS, Free Ce6 and CA-NPs at the dose of 5.0 mg/kg under irradiation or not (0.15 W/cm^2^, 5min) and stained with DCFH-DA. Scale bar: 100 μm. **(C)**. Schematic illustration of therapeutic procedure of CA-NPs. **(D,E)** Tumor growth profiles and corresponding tumor mass (day 21) of the mice bearing MB49 tumors treated with PBS, Free Ce6 and CA-NPs at the dose of 5.0 mg/kg Ce6 under irradiation or not (660 nm, 0.15 W/cm^2^, 5 min). (n = 7; ns: no significance, **p < 0.01, ***p < 0.001).

Taking advantage of preferable photoactivity and targeting ability of CA-NPs, we then sought to investigate the *in vivo* anti-tumor effect against intractable bladder tumors. Firstly, we intravenously injected CA-NPs and free Ce6 at the dose of 5.0 mg/kg into mice bearing MB49 tumor models and the mice were suffered from irradiation at 12 h post-injection. Half an hour before irradiation, DCFH-DA was injected intratumorally at the dose of 10 μM. Then the tumor tissues were pick out at 30 min post-irradiation and were cut into 10 μm slices, followed by staining with Hoechst33342 to measure the *in vivo* ROS production. Both free Ce6 and CA-NPs induced ignorable ROS in the absence of irradiation as evidenced by the undetectable green fluorescence in the tumor sections (Figure 5A), while CA-NPs led to remarkable ROS generation upon irradiation and the amount of ROS was higher than that of free Ce6 group (Figure 5B), suggesting the enhanced tumor accumulation and elevated photoactivity cooperatively contributed to the abundant *in vivo* ROS production.

Next, we further evaluated the *in vivo* therapeutic effect of CA-NPs against subcutaneous MB49 tumor models. Briefly, CA-NPs and free Ce6 (5.0 mg/kg) were intravenously administrated into MB49 tumor model-bearing mice, and the mice were then treated with or without irradiation (660 nm, 0.15 W/cm^2^, 5 min) at 12 h post-injection (Figure 5C). The tumor volume and mice weight were observed and recorded during 21 days as well as the tumor weight. During the observation time, the tumor volume was rapidly increased as compared to that at day 0, and the similar trend was also shown in PBS group upon irradiation, indicating that laser exposure alone didn’t affect the growth of tumor. Meanwhile, the tumor growth profile in both CA-NPs and free Ce6 groups exhibited no significant change as compared to that of PBS group. And upon irradiation, a slight delay of tumor growth was clearly detected in the free Ce6 group, while the tumors were totally disappeared after treatment of CA-NPs under laser exposure during 21 days (Figure 5D). Consequently, the tumor weight was considerably decreased in the group of CA-NPs upon irradiation (Figure 5E). The excellent therapeutic effect of CA-NPs was attributed to the generation of ROS since there was no significant temperature elevation at tumor region *in vivo* (Supplementary Figure S6). In addition, no significant decrease of the body weight was observed during 21 days for those treated mice, indicating that free Ce6 and CA-NPs have negligible side effects on the mice subjected to treatment (Supplementary Figure S7). Collectively, the abundant *in vivo* ROS production induced by CA-NPs via enhanced tumor accumulation and elevated photoactivity confers potent PDT effect against murine bladder tumor models.

### 3.6 Anti-tumor effect of CA-NPs against bladder tumor sections from patients

**FIGURE 6:**
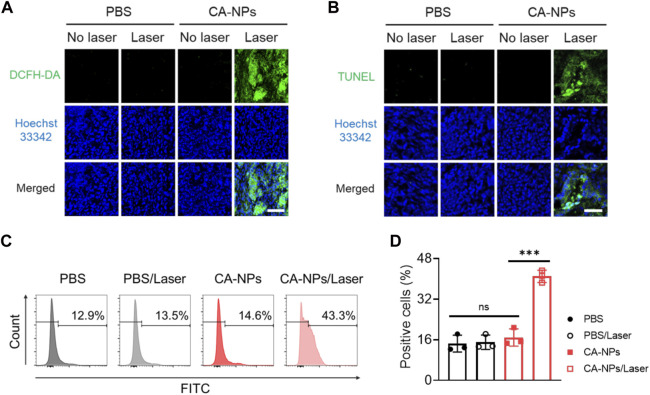
Anti-tumor effect of CA-NPs against bladder tumor sections from patients. **(A)** DCFH-DA staining of human bladder tumor treated with PBS and CA-NPs under irradiation or not (0.15 W/cm^2^, 5min) post intratumoral injection of 1 μg Ce6. Scale bar: 100 μm. **(B)** TUNEL staining of human bladder tumor treated with PBS and CA-NPs under irradiation or not. Scale bar: 50 μm. **(C,D)**. Flow cytometric measurement of positive cells of TUNEL staining in human bladder tumor treated with PBS and CA-NPs under irradiation or not. (n = 3; ns: no significance, **p < 0.01, ***p < 0.001).

To further demonstrated the clinical applicability of CA-NPs, we selected bladder tumor sections from patients and assess the anti-tumor effect. Firstly, the freshly obtained bladder tumor tissues were intratumorally injected with CA-NPs (1 μg) and DCFH-DA, and suffered from 660 nm laser irradiation for 5 min at the density of 0.15 W/cm^2^. Then the tumor tissues were cut into 10 μm slices and stained with Hoechst33342, followed by observation using confocal laser scanning microscopy. As shown in Figure 6A, emerged green fluorescence signals was detected in the tumor tissues treated with CA-NPs under light exposure. On the contrast, tumor tissues in other control groups showed negligible fluorescence signals (Supplementary Figure S8). Moreover, we also applied TUNEL assay to quantitatively measure the apoptosis of bladder tumor section after various treatments. In the group of CA-NPs under irradiation, strong green fluorescence was observed compared to compared to saline and CA-NPs without irradiation (Figure 6B, Supplementary Figure S9). Moreover, 41.0% apoptosis-positive cells were detected and were 2.8-fold and 2.4-fold higher as compared to that of PBS and CA-NPs group without irradiation, respectively (Figures 6C, D), revealing the superior PDT effect from CA-NPs against clinical bladder tumor tissues.

## 4 Conclusion

PDT was clinically approved for the treatment of bladder cancer in 1993 since it is feasible to insert the optical fiber into the urethra, showing the tremendous potential of PDT in the treatment of bladder cancer. To address current challenge of PDT against bladder cancers such as insufficient targeted delivery of photosensitizer, we present the CA-NPs that is accomplished *via* a well-defined precipitation reaction inside albumin nanoreactor for targeted delivery of Ce6 to induce potent PDT effect against murine bladder cancer and human restricted tumor tissues. CA-NPs shows enhanced ROS production and excellent resistance to photobleaching as compared to Ce6. Furthermore, CA-NPs are effectively internalized by cancer cells and allows rapid translocation from lysosome to cytosol to induce potent PDT effect against cancer cells. Owing to the suitable size, CA-NPs show the excellent capacity to accumulate and penetrate in the whole tumor sites. CA-NPs administered intravenously with single dose can generate ROS immediately after irradiation and produce strong anti-tumor efficacy to eradicate difficult-to-treat murine bladder cancer models. Moreover, CA-NPs also induce severe apoptosis of tumor cells in freshly restricted human bladder cancer *via* abundant ROS generation. Therefore, these results indicate HSA as an emerging carrier for cancer treatment with tremendous clinic translational potential to expand the delivery of photosensitizers and other small molecule drugs.

## Data Availability

The raw data of this article will be made available by the authors, without undue reservation.
