# Supplementary material for: Translational albumin nanocarrier caging photosensitizer for efficient cancer photodynamic therapy
Source: Front Bioeng Biotechnol. 2023 Feb 1;11:1132591. doi: 10.3389/fbioe.2023.1132591 (PMC9929546; doi:10.3389/fbioe.2023.1132591)
Supplement: Supplementary file 1 [file Table1.docx]

Supplementary Material

Translational albumin nanocarrier caging photosensitizer for efficient cancer photodynamic therapy

Jie Luo 1†, Zhijun Miao 1,2†, Xinglong Huang 1, Yifan Yang 1, Ming Liu 1, Gang Shen 2*, Tao Yang 1*

^1^Jiangsu Key Laboratory of Neuropsychiatric Diseases, College of Pharmaceutical Sciences, Soochow University, Suzhou, China

^2^Department of Urology, Dushu Lake Hospital Affiliated to Soochow University, Suzhou, China

†These authors contributed equally to this work

*** Correspondence:**T. Yang (tyang0920@suda.edu.cn); G. Shen (gshen119@163.com)

**Keywords: human serum albumin, photosensitizer, target delivery, photodynamic therapy, bladder cancer.**

**Supplementary Figure 1.** The hydrodynamic diameter of CA-NPs in H_2_O, PBS and culture medium during 7-day storage at room temperature.

**Supplementary Figure 2**. Accumulative release of Ce6 from CA-NPs at pH 5.5, pH 6.5 and pH 7.4 buffer within 48 h.

**Supplementary Figure 3.** Temperature elevations of PBS, free Ce6 and CA-NPs at the concentration of 5**.**0 μg/mL Ce6 under irradiation for 5 min (660 nm, 0.15 W/cm^2^).

**
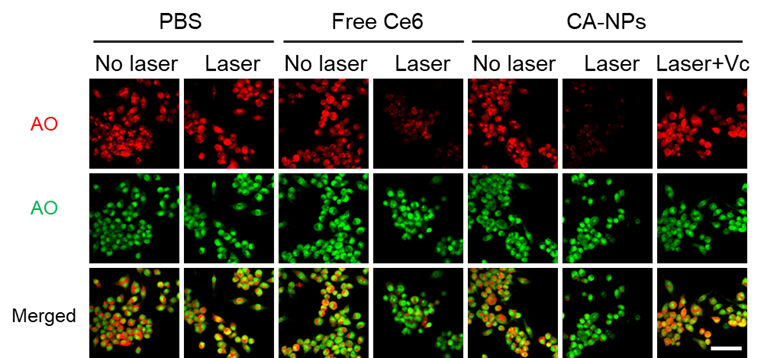
**

**Supplementary Figure 4.** Observation of lysosomal disruption of MB49 cells treated with PBS, free Ce6 and CA-NPs at dose of 5.0 μg/mL Ce6 under irradiation (5 min, 0.15 W/cm^2^) using AO staining. Scale bar: 50 μm. Vitamin C, Vc.

**Supplementary Figure 5.** Measurement of fluorescent intensity of JC-1 staining in MB49 cells. (n = 3; ns: no significance, ****P* < 0.001)

**
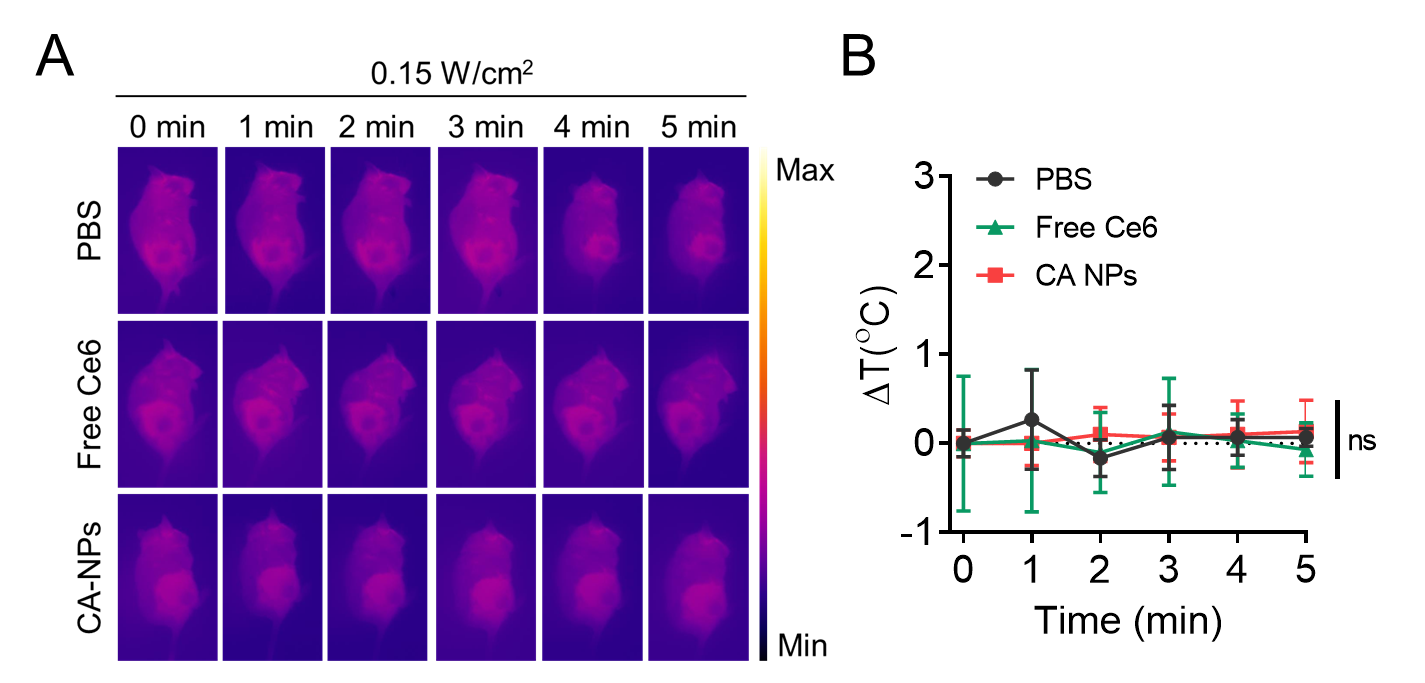
****Supplementary Figure 6.** (A, B) Infrared thermography and temperature elevation of mice bearing MB49 tumor treated with PBS, free Ce6 and CA-NPs in the presence of 5 min irradiation (660 nm, 0.15 W/cm^2^) at the dose of 5.0 mg/kg Ce6.

**Supplementary Figure 7.** Body weight of mice bearing MB49 tumor treated with PBS, PBS plus irradiation, Ce6, Ce6 plus irradiation, CA-NPs and CA-NPs plus irradiation at the dose of 5.0 mg/kg Ce6 during 21 days. (n = 7; ns: no significance)

**Supplementary Figure 8.** Measurement of fluorescent intensity of DCFH-DA in human bladder tumor tissue intratumorally injected with PBS, and CA-NPs under irradiation or not at the dose of 1.0 μg Ce6. (n = 3; ns no significance, ****P* < 0.001).

Supplementary Figure 9. Measurement of fluorescent intensity of FITC of TUNEL in human bladder tumor tissue injected with PBS, and CA-NPs intratumorally under irradiation or not at the dose of 1.0 μg Ce6. (n = 3; ns: no significance, ****P* < 0.001)
